# Factors of Never Screened with Faecal Occult Blood Test in Public Primary Care Facilities

**DOI:** 10.31557/APJCP.2021.22.1.163

**Published:** 2021-01

**Authors:** Mohd Fazeli Sazali, Syed Sharizman Syed Abdul Rahim, Richard Avoi, Mohd Rohaizat Hassan, Firdaus Hayati, Zahir Izuan Azhar, Mohammad Saffree Jeffree, Khamisah Awang Lukman, Naing Oo Tha, Helmy Sajali, Azman Atil, Muhammad Aklil Abd Rahim

**Affiliations:** 1 *Department of Community Health and Family Medicine, Faculty Medicine and Health Sciences, Universiti Malaysia Sabah, Malaysia. *; 2 *Department of Community Health, Faculty of Medicine, Universiti Kebangsaan Malaysia, Malaysia. *; 3 *Department of Surgery, Faculty of Medicine and Health Sciences, Universiti Malaysia Sabah, Kota Kinabalu, Malaysia. *; 4 *Department of Public Health Medicine, Faculty of Medicine, Universiti Teknologi MARA, Malaysia. *

**Keywords:** Faecal occult blood test, factors, predictors, colorectal cancer, never screened

## Abstract

**Background:**

Colorectal cancer (CRC) is still a major public health threat. In the effort to reduce CRC incidence and mortality, faecal occult blood test (FOBT) is currently the screening tools used for early detection of CRC. However, the uptake of FOBT screening is less than promising. This study aims to identify the prevalence and predictors of Never Screened with FOBT (NS-FOBT).

**Methods:**

A cross sectional study was conducted in five health clinics under Kota Kinabalu district, Sabah, Malaysia Borneo involving 162 attendees with age of 50 years old and above. A validated self-administered questionnaire was used to collect the data. Multiple logistic regression analysis was used to determine the predictors of NS-FOBT.

**Results:**

The prevalence of NS-FOBT was 85.8% (n=139). Important predictors of NS-FOBT were age (aOR: 0.922; 95% CI: 0.855, 0.995; p=0.035), Bumiputera ethnicity (vs Non Bumiputera; aOR: 4.285; 95% CI: 1.384, 13.263; p=0.012), knowledge score (aOR: 0.921; 95% CI: 0.856, 0.99; p=0.027), and attitude score (aOR: 0.801; 95% CI: 0.702, 0.913; p=0.001).

**Conclusion:**

There is high prevalence of NS-FOBT. Age, ethnicity, knowledge, and attitude were important predictors of NS-FOBT. Strategies are needed to improve FOBT screening rate among the public. Socio-culturally tailored health promotion strategies as well as strengthening the communication, collaboration, and education to enhance the role of family physician is vital in improving the CRC prevention and care.

## Introduction

Colorectal cancer (CRC) is becoming the most common cancer in Asia and worldwide with estimation of 10.9% of all cancer death among men and 9.5% among women (International Agency for Research on Cancer (IARC) 2018). Meanwhile, CRC is the second most common cancer, accounting for 13.5% from all types of cancer, after breast cancer (19.0%) among Malaysian population. In addition, more than 70% of CRC cases were detected at late stage (Azizah et al., 2019). It was found that 5 years survival of CRC in Malaysian patients is about 40%, which is lower than the high income countries such as Singapore (60%) and South Korea (73%) (Veettil et al., 2017). Furthermore, malignant tumour is among five top causes of death among Malaysian and increasing in trend for the past 20 years, accounts for 8.6% on 1996 and 13.6% deaths on 2015 in Malaysians hospitals (Ministry of Health Malaysia 2017).

Mortality and morbidity of CRC are highly preventable, provided that the diagnosis and treatment were made early. Most of CRC cases appear to develop from benign and precancerous polyps, where the incidence and mortality of CRC can be reduced by performing early screening through removal of adenomatous polyps and sessile serrated polyps (Simon, 2016). One of the recommended screening tools is by using faecal occult blood test (FOBT). Early screening using guaiac faecal occult blood test (gFOBT) is able to reduce CRC mortality by 8-16% (Gini et al. 2020). United States Preventive Service Task Force (USPSTF) recommended screening for CRC using colonoscopy, sigmoidoscopy or FOBT testing starting at the age of 50 until 75 years old. Whereby, Asia Pacific Consensus on CRC screening recommends starting age for screening at 50 years old, thus FOBT in resource limited settings and screening should be made as priority especially in Asian countries (Sung et al., 2008). 

Recommendation by European Commission states that the minimum uptake of CRC screening among average risk group is 45% and 65% as the optimum rate (Vieth et al., 2012). Despite the current evidence and presence of specific guidelines in the CRC screening, the prevalence of never screened for CRC was varied and relatively high. Studies in various countries shown relatively high proportion of never had CRC screening such as in Israel (98.8%) (Vinker et al. 2002), Canada (76.5-90.4%) (Tinmouth et al. 2015; Zarychanski et al. 2007), United States (90.1%) (Ioannou et al., 2003), South Korea (25% in 2012) (Choi et al., 2012), Thailand (37.1%) (Khuhaprema et al. 2014), and Malaysia (97-99.3%) (Koo et al., 2012; Yusoff et al., 2012). 

Previous study found that various factors could affect people’s decision to not screen for FOBT, such as sociodemographic and socioeconomic background (Brenner et al., 2015; Lin et al., 2017), health access factor (Harada et al., 2017; Ioannou et al., 2003), preventive behaviour (Bernardo et al., 2018), as well as knowledge and attitude regarding CRC (Christou and Thompson 2012; Douma et al., 2018; Rosli et al., 2017; Su et al., 2013; Sung et al., 2008). 

The role of primary care in cancer detection and management has been increasing in their importance and demand among the public, as the primary care physician serve as a gate keeper in healthcare service delivery. A qualitative study conducted among cancer patients mentioned about their preference to primary care as it provides more convenience and less waiting time, compared to hospital-based care (Idris et al., 2020). Thus, this shows that primary care has a major role in the cancer patient management. Hence, the objective of this study is to identify the prevalence of Never Screened with FOBT (NS-FOBT) and its associated predictors among attendees in public health clinics in Kota Kinabalu, Sabah, Malaysia Borneo. These findings are useful to guide both the public health physicians and clinicans in order to develop effective intervention strategies to improve the uptake of FOBT screening. 

## Materials and Methods

*Study design, location and population*

A cross sectional study was conducted from January until August 2020, located in five health clinics in Kota Kinabalu, Sabah, which were Inanam Health Clinic, Luyang Health Clinic, Menggatal Health Clinic, Telipok Health Clinic and Likas Health Clinic. The study location is situated in Kota Kinabalu, which is the state capital and one of the districts in Sabah. Sabah is one of states in Malaysia and located at North of Borneo Island. The study population is among attendees at the health clinic, with age of 50 years old and above. Those with any mental health problem, have been diagnosed with CRC and not consented to be a study subject were excluded from the study. Proportionate stratified random sampling was used to determine the sampling population for each of the health clinic, which is based on the daily attendance of patients with age 50 and above. Subsequently, on the day of data collection, the study subject is selected using systematic random sampling where every 5th patients were selected, according to their calling number that was given by health clinic staff.

*Research tools*

A validated English and Malay version of questionnaire was used for data collection, based on the previous study (Harmy et al., 2011). The questionnaire consists of six sections, which are: Sociodemographic and socioeconomic background, characteristics related to health or health access, preventive behaviour, knowledge and attitude towards CRC, as well as FOBT screening status. The Cronbach alpha for knowledge domain was 0.65 and attitude was 0.82. There were 29 questions for knowledge section and 10 questions in attitude section. A Likert scale of 5 (strongly agree / agree / neutral / disagree / strongly disagree) were used for knowledge and attitude items. Scores of ‘5’, ‘4’, ‘3’, ‘2’ and ‘1’ were used for correct or positive items and were reversed for the incorrect or negative items. For knowledge domain, minimum score is 29 and maximum score is 145. For attitude domain, minimum score is 10 and maximum score is 50. Each of total score was transformed into percent score. Previous author set the cut off value at 80%, where the score of 80% and above is considered as good knowledge, while score below than 80% is considered as poor knowledge.

In the knowledge section, the respondents were asked regarding epidemiology, risk factors, sign and symptoms, CRC treatment and screening modalities. Meanwhile, in the attitude section, the respondents were asked regarding their perceived susceptibility to CRC, personal belief that CRC is preventable, personal belief on effectiveness of traditional medicine to treat CRC, perceived benefit to prevent CRC by eating vegetables and regular exercise, perceived benefit to gain more information regarding CRC, self-efficacy to spend time to get screened in order to prevent CRC, personal belief that screening is beneficial for health, perceived barrier to the screening due to absence of CRC symptoms, and personal belief of vitamin E could effectively prevented CRC. 

Finally, in the last section, FOBT screening status was assessed. The respondents were asked, whether they had any previous test done in their lifetime, which require them to send stool sample to check for blood in the stool, for CRC screening. Prior to the data collection, the questionnaire was pre-tested among randomly selected 30 individuals resembles the study population, to test for face validity of the instrument.

*Data analysis*

The data was analysed using International Business Machine Statistical Program for Social Sciences (IBM SPSS) version 26. The normality of continuous numerical data was determined using histogram, Q-Q plot, boxplot, and Shapiro Wilk test. 

The relationship of each independent categorical variable with the FOBT screening status were determined using simple logistic regression analysis. P-value less than 0.05 is considered statistically significant. Variables with p-value less than 0.25 from the simple logistic regression analysis were subjected for multivariate binary logistic regression analysis. The cut-off point of p-value at 0.25 was recommended because the use of traditional levels such as 0.05 often fails to identify variables known to be important (Bursac et al. 2008). Multivariate logistic regression analysis was used to determine the independent factors associated with NS-FOBT. 

*Ethical consideration*

The ethical approval for this study was obtained from Medical Research Ethic Committee, Universiti Malaysia Sabah (Approval Code: JKEtika 1/20 (1)) and Medical Research and Ethics Committee, Ministry of Health Malaysia (Ethics Initial Approval: NMRR-19-3005-51437(IIR)). Furthermore, the permission to conduct the study in public health clinic also was obtained from Sabah State Health Director (reference: JKNS/KA/10/01/737(19)). 

## Results

*Characteristics of respondents*

A total of 162 respondents participated in this study. Respondents’ age was normally distributed with the mean age of 61.27 (SD: 7.22). Majority were male (51.2%), Bumiputera ethnicity (consist of Malays, indigenous group in East Malaysia, and natives) (77.8%), married (77.8%), secondary school as the highest education (44.4%) and currently employed (38.9%). Median monthly household income was RM1054.00 (IQR: 2138) and majority have income RM1160 and below (52.5%). Meanwhile, 88.9% of respondents reported time to travel from home to regular clinic is 30 minutes and below, with median duration was 15 minutes (IQR: 20), and majority had routine visit to their doctor less than one year (77.2%). Upon assessing the preventive behaviour, majority reported had any exercise activity in past 2 weeks (56.2%) and not a current smoker (79%). In terms of assessment of knowledge regarding CRC, majority had poor knowledge score (97.5%). The knowledge score was normally distributed with mean score of 68.42 (SD: 5.96). Meanwhile, majority had poor attitude score (77.8%). The attitude score was found to have normal distribution with the mean score of 71.79 (SD: 9.63).

*Prevalence of NS-FOBT*

The overall prevalence of NS-FOBT was 85.8% (n=139). The average age of respondents who NS-FOBT was 60.83 (SD: 7.29), compared to those who were ever screened for FOBT (mean age: 63.87 [SD: 6.31]). The highest prevalence of NS-FOBT were among females (91.1%), Bumiputera (90.5%), currently not living with spouse (91.7%), never had formal education (97.3%), in labour force (88.5%), monthly household income RM1160 and below (90.6%) (see Table 1). In terms of factor related to health access and preventive behaviour, those who took longer than 30 minutes to regular health clinic (94.4%), last visit to doctor more than one year (97.3%), currently exercise (86.8%), and currently smoking (91.2%) (see Table 2). Majority reported never had FOBT screening had poor knowledge score (86.7%) and poor attitude score (92.9%) with lower average knowledge and attitude score (see Table 3).

*Factors and predictors for NS-FOBT*

From the analysis, there are several independent variables that were significantly associated with NS-FOBT such as Bumiputera ethnicity (vs Non Bumiputera; Crude OR [cOR]: 4.18; 95% CI: 1.657, 10.548; p<0.01) (Table 1), knowledge score regarding CRC (cOR: 0.884; 95% CI: 0.831, 0.941; p<0.001), and attitude score regarding CRC (cOR 0.789, 95% CI: 0.706, 0.882; p<0.001) (Table 3). However, there were other independent variables that was found to have no significant association with NS-FOBT, such as age (p=0.066), gender (p=0.063), marital status (p=0.262), monthly household income (p=0.072), employment status (p=0.232), time of travel from house to usual public health clinic (p=0.288), interval from the last visit to doctor or health clinic (p=0.05), ever exercised in the past two weeks (p=0.677), and current smoking status (p=0.319).

There were nine independent variables with p-value <0.25 out of thirteen variables were analysed using multiple logistic regression. These variables include age (P= 0.066), gender (p= 0.063), ethnicity (p= 0.002), monthly household income (p= 0.072), education background (p=0.05), employment status (p= 0.232), interval since respondents’ last visit to doctor or public health clinic (p= 0.05), knowledge score (p<0.001) and attitude score (p<0.001). However, from the nine variables selected, only four variables were identified as the best final model for the multivariate analysis which were age, ethnicity, knowledge score, and attitude score. The significant predictor for NS-FOBT were age (adjusted OR [aOR]: 0.922; 95% CI: 0.855, 0.995; p=0.035), Bumiputera ethnicity group (vs Non Bumiputera; aOR: 4.285; 95% CI: 1.384, 13.263; p=0.012), knowledge score (aOR: 0.921; 95% CI: 0.856, 0.99; p=0.027), and attitude score (aOR: 0.801; 95% CI: 0.702, 0.913; p=0.001).

**Table 1:** Demographic and Socioeconomic Factors and Predictors for NS-FOBT

Sociodemographic	FOBT screening		cOR (95% CI)	p-value	aOR (95% CI)	p-value
	Screened, n (%)	Never screened, n (%)				
Overall prevalence	23 (14.2)	139 (85.8)				
Age						
Mean (SD)	63.87 (6.31)	60.83 (7.29)	0.945 (0.89 - 1.004)	0.066	0.922 (0.855 -0.995)	0.035*
Gender						
Male	16 (19.3)	67 (80.7)				
Female	7 (8.9)	72 (91.1)	2.456 (0.951 - 6.341)	0.063		
Ethnicity						
Non-bumiputera	11 (30.6)	25 (69.4)				
Bumiputera	12 (9.5)	114 (90.5)	4.180 (1.657 - 10.548)	0.002*	4.285 (1.384 - 13.263)	0.012*
Marriage status						
Currently living with spouse	20 (15.9)	106 (84.1)				
Currently not living with spouse	3 (8.3)	33 (91.7)	2.075 (0.580 - 7.426)	0.262		
Education level						
Had formal education	22 (17.6)	103 (82.4)				
No formal education	1 (2.7)	36 (97.3)	7.689 (1.000 - 59.113)	0.05		
Employment						
In labour force	11 (11.5)	85 (88.5)				
Not in labour force	12 (18.2)	54 (81.8)	1.717 (0.708 - 4.166)	0.232		
Monthly household income						
More than RM1160	15 (19.5)	62 (80.5)				
RM1160 and below	8 (9.4)	77 (90.6)	2.329 (0.927 - 5.849)	0.072		

(*), Significant, p<0.05; ^a^Never-Screened-for-FOBT (NS-FOBT)

**Table 2:** Factors Related to Health and Health Access and Preventive Behaviour with NS-FOBT

Sociodemographic	FOBT screening		cOR (95% CI)	p-value	aOR (95% CI)	p-value
	Screened, n (%)	Never screened, n (%)				
Time to travel from house to usual PHC						
30 minutes and less	22 (15.3)	122 (84.7)				
More than 30 minutes	1 (5.6)	17 (94.4)	3.066 (0.388 - 24.227)	0.288		
Interval since last visit to doctor/ health clinic						
1 year and less	22 (17.6)	103 (82.4)				
More than 1 year	1 (2.7)	36 (97.3)	7.689 (1.000 - 59.113)	0.05		
Exercise status						
Yes	12 (13.2)	79 (86.8)				
No	11 (15.5)	60 (84.5)	0.829 (0.342-2.006)	0.677		
Smoking status						
Current smoker	3 (8.8)	31 (91.2)				
Not current smoker	20 (15.6)	108 (84.4)	0.523 (0.146 - 1.875)	0.319		

Note: (*) = Significant, p<0.05; ^a^Never-Screened-for-FOBT (NS-FOBT)

**Table 3:** Knowledge and Attitude Predictors for NS-FOBT

Sociodemographic	FOBT screening		cOR (95% CI)	p-value	aOR (95% CI)	p-value
	Screened, n (%)	Never screened, n (%)				
Knowledge level (%)						
Mean (SD)	73.37 (6.20)	67.60 (5.53)				
Poor	21.00 (13.3)	137 (86.7)				
Good	2.00 (50.0)	2 (50.0)	0.884 (0.831 - 0.941)	<0.001*	0.921 (0.856 - 0.99)	0.027*
Attitude level						
Mean (SD)	80.17 (9.67)	70.40 (8.92)				
Poor	9.00 (7.1)	117 (92.9)				
Good	14 (38.9)	22 (61.1)	0.789 (0.706 - 0.882)	<0.001*	0.801 ( 0.702 - 0.913)	0.001*

Note: (*) = Significant, p<0.05; ^a^Never-Screened-for-FOBT (NS-FOBT)

## Discussion

The prevalence of NS-FOBT reported in this study was higher compared to several observations in various countries such as in England, Canada, and United States (Moss et al., 2017; Zarychanski et al., 2007). These countries adopted nationwide biennial FOBT screening in national screening recommendation. Meanwhile, in Malaysia, the country recommended selective opportunistic screening approach, which only targeted those attending public health clinic as screening subject (Ministry of Health, 2015). Narrow target group in Malaysian CRC screening strategy explained higher never screened for FOBT in current study. In addition, elderly who are in the eligible group for CRC screening might have several barriers to perform CRC screening due to financial situation, inadequate design and availability of hospital care services, lack of long term care facilities, and increased waiting time for healthcare (Doetsch et al., 2017). However, the prevalence of non-responder in CRC screening is lower in a pilot cross sectional study in Malaysia where there was only 5.3% defaulter rate for first round and 9.4% defaulter rate for second round of invitation (Hassan et al., 2016). Contradicting prevalence might be due to the nature of pilot program of the study, where there is a possibility that the known barrier for screening might be adjusted in order to ensure the success of the pilot program. Even though prevalence of CRC screening uptake in current study (14.5%) was higher compared to uptake level from previous study in Malaysia (Koo et al., 2012; Yusoff et al., 2012), it still has not reached the recommended level of CRC screening uptake (Vieth et al., 2012). 

People at younger age were more likely to not participate in FOBT screening compared to the older age. This result was consistent with several studies (Lin et al., 2017; Lo et al., 2015). Better cancer screening participation among older age group might be related to more positive attitude towards cancer screening, compared to the younger age group (Cullati et al., 2009). Furthermore, past study in US found older age group were more likely to be screened due to implementation of National Health Insurance Programme which covers health related expenses including CRC screening for US citizens aged 65 and above. However, this is different in current study context, as most of health-related expenses in government health clinic were largely subsidised by government, including FOBT screening. 

This study had identified that Bumiputera ethnicity was a significant independent factor that predicted the NS-FOBT. Bumiputera group were found to have four times higher odds to be more likely to not participate in FOBT screening compared to Non-Bumiputera group. This finding might be influenced by low level of adequate health literacy among Bumiputera group (Karim, 2020). The finding also supported by a nationwide survey in United States, which concluded that white population have higher level of unhealthy behaviour than black and/or Hispanic populations, particularly for smoking, second hand smoke exposure, and inadequate cancer screening (Anderson et al., 2004).

This study also identified that knowledge level regarding CRC was associated with FOBT screening status. Respondents who have better knowledge in CRC will more likely to have FOBT screening compared to those who have poor knowledge, where the findings remain the same after adjusted for other confounding variables. The similar finding was also reported in several studies (Christou, and Thompson, 2012; Su et al., 2013). An observation among indigenous group in Australia, reported that those with medium to high knowledge score were 8.5 to 10 times more likely to consider CRC screening using FOBT (Christou and Thompson, 2012). The possible reason is that individuals with adequate knowledge regarding CRC are more likely to identify warning signs and more likely to participate in appropriate health seeking behaviour, compared with those with inadequate knowledge (Su et al., 2013).

Advanced statistical analysis in this study found that attitude score was a significant predictor to NS-FOBT, where every unit increase in attitude score has lowered the odds for not screened for FOBT by 19.9%. The finding is consistent with several studies done ine the past (Naing et al., 2014; Sung et al., 2008). Attitude score which generally assessed on several constructs of Health Belief Model (HBM), concluded that better attitude score reduced the likelihood to not screen for FOBT. The finding is supported by a study among population in Hong Kong where all the construct in HBM (except cues for action), were found to have significant association with the uptake of CRC screening test (Sung et al., 2008). The findings could be explained by Health Belief Model, which contributes to the key construct in attitude variable, where the model suggested that a person’s belief on personal threat to the particular disease together with personal belief in the benefit of preventive behaviour will predict the likelihood of the person to adopt the recommended behaviour. 

This public health issue may benefit by smart healthcare delivery to improve access via digital health integration. Screening and referral of colorectal cases for colonoscopy can be expedited (Jeffree et al., 2020). One of the strengths of this study is, this study is one of the first published report that explored on various factors that influence the FOBT screening especially in Sabah and Malaysia that is diverse in culture and belief. The main limitation of this study was the study is cross sectional, where the association between predictors and outcome variable may not be taken as an indication of causality as it only measured at a single time point and reverse causality is possible. 

In conclusion, there was high prevalence of NS-FOBT observed among the respondents. Age, ethnicity, knowledge and attitude level regarding CRC were found to be the important predictors for NS-FOBT among the respondents. The findings could guide the public health physician and policy maker to develop new strategy to improve the uptake FOBT screening among public. The strategies include improving health promotion activities to increase the awareness among public, specifically socio-culturally tailored program. Strengthening the communication, collaboration, and further education to enhance the role of family physician is vital in improving the CRC prevention and care. 
